# Fruit and vegetable intake and cardiovascular risk factors in people with newly diagnosed type 2 diabetes

**DOI:** 10.1038/ejcn.2016.180

**Published:** 2016-10-19

**Authors:** M J E Lamb, S J Griffin, S J Sharp, A J M Cooper

**Affiliations:** 1 MRC Epidemiology Unit, University of Cambridge School of Clinical Medicine, Institute of Metabolic Science, Cambridge, UK; 2 The Primary Care Unit, Institute of Public Health, University of Cambridge, Cambridge, UK

## Abstract

**Background/Objectives:**

The cardiovascular benefit of increasing fruit and vegetable (F&V) intake following diagnosis of diabetes remains unknown. We aimed to describe how quantity and variety of F&V intake, and plasma vitamin C, change after diagnosis of type 2 diabetes and examine whether these changes are associated with improvements in cardiovascular risk factors.

**Subjects/Methods:**

A total of 401 individuals with screen-detected diabetes from the ADDITION-Cambridge study were followed up over 5 years. F&V intake was assessed by food frequency questionnaire and plasma vitamin C at baseline, at 1 year and at 5 years. Linear mixed models were used to estimate associations of changes in quantity and variety of F&V intake, and plasma vitamin C, with cardiovascular risk factors and a clustered cardiometabolic risk score (CCMR), where a higher score indicates higher risk.

**Results:**

F&V intake increased in year 1 but decreased by year 5, whereas variety remained unchanged. Plasma vitamin C increased at 1 year and at 5 years. Each s.d. increase (250g between baseline and 1 year and 270g between 1 and 5 years) in F&V intake was associated with lower waist circumference (−0.92 (95% CI: −1.57, −0.27) cm), HbA_1c_ (−0.11 (−0.20, −0.03) %) and CCMR (−0.04 (−0.08, −0.01)) at 1 year and higher high-density lipoprotein (HDL)-cholesterol (0.04 (0.01, 0.06) mmol/l) at 5 years. Increased plasma vitamin C (per s.d., 22.5 μmol/l) was associated with higher HDL-cholesterol (0.04 (0.01, 0.06) mmol/l) and lower CCMR (−0.07 (−0.12, −0.03)) between 1 and 5 years.

**Conclusions:**

Increases in F&V quantity following diagnosis of diabetes are associated with lower cardiovascular risk factors. Health promotion interventions might highlight the importance of increasing, and maintaining increases in, F&V intake for improved cardiometabolic health in patients with diabetes.

## Introduction

Type 2 diabetes is a leading cause of premature morbidity and mortality, much of which can be explained by the increased risk of cardiovascular disease (CVD).^1, 2^ Previous studies have shown that adhering to specific dietary patterns such as the Dietary Approaches to Stop Hypertension diet (DASH-diet) or the Mediterranean Diet can lower the risk of developing CVD, even among those initially at high such as, individuals with diabetes.^3, 4, 5^ A common theme underpinning these diets is an emphasis on consuming more fruits and vegetables (F&V). A large number of studies have demonstrated independent health benefits of a diet rich in F&V,^6, 7, 8^ findings that are supported by several studies that examined associations using plasma vitamin C as an objective biomarker of F&V intake.^9, 10^ More recently, variety of F&V intake, independent of quantity, has been considered in relation to risk of diabetes and CVD.^11, 12, 13^ To our knowledge, no studies have examined the relationship between repeat measures of quantity and variety of F&V intake and CVD risk factors among individuals with diabetes over 5 years of follow-up, corroborated using plasma vitamin C levels.

Using data from the Anglo–Danish–Dutch Study of Intensive Treatment In People with Screen-Detected Diabetes in Primary Care (ADDITION)-Cambridge study, we examined the longitudinal relationship between quantity and variety of F&V intake and plasma vitamin C levels with CVD risk factors in participants with diabetes who were followed up for 5 years.

## Methods

### Study design

The design and rationale for the ADDITION-Cambridge study have been reported in detail elsewhere.^14^ In brief, individuals were recruited from 49 general practice clinics in the East of England, UK, for a stepwise diabetes screening programme. Diabetes was diagnosed according to WHO criteria.^15^ Eligible individuals were aged 40–69 years with no known diabetes and were within the top 25% of a diabetes risk score.^14, 16^ Exclusion criteria included being pregnant or lactating, having an illness with a prognosis of death within 1 year or a psychiatric illness that was likely to preclude involvement or informed consent. Of the 33 539 individuals who were invited to attend screening, 867 were identified to have diabetes and agreed to participate in the randomised control trial. The aim of the trial was to compare intensive treatment of multiple risk factors with routine care in individuals with screen-detected diabetes. Participants were cluster-randomised by general practice clinic. In the intensive treatment group, practitioners were encouraged to follow a stepwise target-led treatment regimen to reduce and control CVD risk factors including blood glucose and lipid levels. This group additionally received theory-based health promotion materials including encouragement to consume at least five portions of F&V per day. The routine care group received care, which followed UK national guidelines for diabetes management.^17^ Participants attended for follow-up health assessments after 1 and 5 years. As there was no interaction by trial group, we pooled both trial groups and conducted a cohort analysis.

Ethical approval was obtained from the Eastern Multi-Centre Research Ethics Committee (reference number 02/5/54), and all participants gave written informed consent. The ADDITION-Cambridge trial is registered as ISRCTN86769081.

### Fruit and vegetable intake and plasma vitamin C levels

Plasma vitamin C, an objective biomarker of F&V intake,^9, 18^ was measured using a Fluoroskan Ascent FL fluorometer. Self-reported F&V intake was assessed using a validated 130-item food frequency questionnaire (FFQ).^19^ Participants were asked to report the frequency of food consumption on a nine-point scale ranging from ‘never or less than once per month' to ‘more than six times per day'. Variety of F&V intake was derived by summing the total number of unique fruit and vegetable items consumed at least once per week. Possible variety ranged from 0 to 37 items. Quantity of F&V intake was derived by summing the total quantity (in grams/day) of different F&V consumed over the period of 1 week, divided by seven to quantify daily intake. We did not include potatoes in our analyses, as they differ from vegetables in terms of energy and carbohydrate content and are commonly substituted for cereals rather than vegetables.^20^ We also did not include fruit juice, as it is not considered to be equivalent to whole fruit regarding fibre content and satiety value.^21^

### Measurement of cardiovascular risk factors

Baseline, 1 year and 5 year health assessment visits to the study clinic included clinical and anthropometric measures. HbA_1c_ was measured in venous samples using an ion-exchange high-performance liquid chromatography method (Tosoh Bioscience, Redditch, UK). Serum total cholesterol, high-density lipoprotein (HDL)-cholesterol and triglycerides were measured in non-fasted samples using enzymatic techniques (Dade Behring Dimension analyser, Newark). Blood pressure was determined based on the mean of three measurements performed after 10 min of rest, while participants were seated with a cuff placed on their predominant arm at the level of the heart, using an automated sphygmomanometer (Omron M4, UK). Height and weight were measured in light clothing, without shoes, using a fixed rigid stadiometer and scale (SECA, UK). Waist circumference was derived based on the mean of two measurements taken with a tape measure halfway between the lowest point of the rib cage and the anterior superior iliac crest while standing.

Clustered cardiometabolic risk scores (CCMR) were derived for all clinic visits by averaging standardised values for waist circumference, systolic blood pressure, HbA_1c_, the natural log of triglycerides and the inverse of HDL-cholesterol. Variables were standardised by subtracting from them sex-specific population means and dividing by sex-specific s.d.'s. Means and s.d.'s at baseline were used to standardise all follow-up CCMR scores. A lower score therefore indicates lower risk.

### Covariates

Self-report questionnaires were used to obtain information on age, sex, occupation and ethnicity. Occupational social class was defined according to the Registrar General's occupation-based classification and comprised three categories: ‘professional, managerial and technical', ‘skilled – manual and non-manual' and ‘partly skilled or unskilled'. Total energy and alcohol intake were assessed using an FFQ.^19^ Time spent in moderate-to-vigorous physical activity was assessed by self-report using the previously validated European Prospective Investigation into Cancer-Norfolk physical activity questionnaire (EPAQ-2).^22^ Medication use was assessed using a self-report questionnaire adapted from the Aberdeen Health Service Research Unit questionnaire.^14^ Very few people reported taking multivitamin supplements; therefore, this was not included in the analysis.

### Statistical analysis

Descriptive characteristics at baseline and at 1 and 5 years of follow-up were summarised using means and s.d.'s or frequencies and percentages. *t*-tests or *χ*^2^ tests were used to examine differences in participant characteristics between those included for these analyses and those excluded because of missing data.

Linear mixed models, with participant-specific random intercepts, were used to estimate the associations between each s.d. increase in change in quantity of F&V intake from baseline to 1 year, and from 1 to 5 years, with CVD risk factor levels and CCMR at 1 and 5 years, respectively. Models were adjusted for age and sex (model 1), exposure and outcome at baseline or 1 year (where applicable), intervention group, occupational social class, smoking status, alcohol intake, total energy intake and self-reported moderate-to-vigorous physical activity at each baseline and follow-up (model 2). We additionally adjusted for use of blood pressure-lowering, lipid-lowering and glucose-lowering medications at each baseline and follow-up in model 2, as appropriate. The same approach was used to examine the associations for variety of F&V intake, as well as for plasma vitamin C levels. Associations of quantity of intake were adjusted for variety and vice versa.

We assessed for interaction of each exposure with sex in model 2. To examine whether associations were mediated by changes in waist circumference (when waist circumference was not the outcome), we additionally adjusted for changes in waist circumference in model 2. We also examined whether associations with CCMR were primarily driven by an association with waist circumference by generating a CCMR score excluding waist circumference (CCMR_•excluding•waist_) and with additional adjustment for waist circumference.

### Sensitivity analyses

As the main analyses were limited to individuals who had data for all variables included in the CCMR score, we also repeated all analyses for each of the cardiometabolic risk factors independently by including the largest number of participants with data for that risk factor. To explore the impact of missing covariate data on our results, we also used multiple imputation by chained equations. For each exposure–outcome relationship, 10 imputed data sets were created, and parameter estimates were combined using Rubin's rules. Each imputation model included both the outcome of interest and all covariates in the analysis models.

All statistical analyses were performed using Stata/SE 13.1 (Stata-Corp, College Station, TX, USA).

## Results

In total, 603 individuals attended all three clinic visits, of whom 401 had complete data for F&V intake, cardiometabolic risk factor levels and covariates (Supplementary Figure 1). A total of 177 individuals were in the intensive treatment trial group and 224 in the routine care trial group. The mean age of the study participants was 61.4 (s.d. 6.6) years at baseline. Men comprised 57% of the cohort (Table 1). Participants with missing follow-up data tended to have a larger waist circumference and higher HbA_1c_ levels at baseline compared with those with complete data. Those with missing data also reported consuming a lower quantity and variety of F&V and had lower plasma vitamin C levels at baseline and at one and five years of follow-up.

As shown in Table 1, intake of F&V increased in both men and women over the first year of follow-up but decreased between 1 and 5 years. The most commonly eaten fruits were apples, oranges and bananas, and the most commonly eaten vegetables were carrots, peas, tomatoes and green salad. Variety of F&V intake remained unchanged over 1 and 5 years of follow-up. Plasma vitamin C levels increased across follow-ups. Quantity and variety of F&V intake were moderately correlated at each time point (*r*=0.54, 0.45 and 0.40 at baseline, 1 and 5 years, respectively). There was a weak correlation between quantity of vegetable intake and plasma vitamin C (*r*=0.10–0.19, at all time points) and a slightly stronger correlation between fruit and combined fruit and vegetable intake and plasma vitamin C (*r*=0.24–0.30, at all time points).

There was no suggestion of interaction by sex (*P*>0.05), and thus all results are presented for men and women combined. Each s.d. change in quantity of F&V intake between baseline and 1 year (250 g) was independently associated with a 0.92 (95% CI: 0.27, 1.57) cm lower waist circumference, a 0.11 (0.03, 0.20) % lower HbA_1c_ level and a 0.04 (0.01, 0.08) lower CCMR score at 1 year (Table 2). Except for HDL-c, F&V intake was not associated with any other CVD risk factor between 1 and 5 years. Change in the quantity of fruit intake (per s.d., 192 g) was associated with a lower waist circumference (−0.89 (−1.56, −0.23) cm), HbA_1c_ level (−0.12 (−0.20, −0.03) %) and CCMR (0.04 (0.01, 0.08)) at 1 year, as well as lower triglyceride levels (−0.10 (−0.19, −0.01) mmol/l) and higher HDL-c (0.03 (0.01, 0.05) mmol/l) at 5 years (per s.d., 197 g). In contrast, change in quantity of vegetable intake (per s.d., 151 g) was associated with a 1.89 (0.29, 3.48) mm Hg lower systolic blood pressure at 1 year. Increases in plasma vitamin C were not associated with any of the CVD risk factors or with CCMR between baseline and one-year (Table 2). Between 1 and 5 years, however, each s.d. increase in plasma vitamin C (22.5 μmol/l) was associated with lower triglyceride levels (−0.11 (−0.21, −0.01 mmol/l), CCMR (−0.07 (–0.12, –0.03)) and with higher HDL-c levels (0.04 (0.01, 0.06) mmol/l).

Additional adjustment for waist circumference had no effect on the observed associations. Associations between change in combined F&V intake and fruit intake separately with CCMR_•excluding•waist_ were not statistically significant between baseline and 1 year but were significant between 1 and 5 years. The results were similar after adjustment for waist circumference in the CCMR_•excluding•waist_ model. The associations between change in plasma vitamin C and CCMR_•excluding•waist_ did not differ from the CCMR score including waist circumference (data not shown).

As shown in Table 3, changes in variety of F&V intake combined and separately were not associated with any of the individual cardiometabolic risk factors or CCMR at 1 or 5 years.

Our findings remained unchanged when we included all participants who had complete data for the cardiometabolic risk factor being analysed (data not shown) and when we performed the analyses following multiple imputation of covariate data (data not shown).

## Discussion

We demonstrate that although patients with screen-detected diabetes tend to increase the quantity of F&V they consume in the first year following diagnosis of diabetes, this increase is not the result of a change in the variety of F&V consumption, and it is not maintained long-term. Nevertheless, we show, for the first time, that even modest increases in F&V intake are associated with clinically meaningful improvements in a number of important CVD risk factor levels, namely waist circumference, HbA_1c_ and HDL-cholesterol. Increased vegetable intake is associated with improved systolic blood pressure, whereas increased fruit intake is associated with improved triglyceride levels. These findings are corroborated by the inverse association between change in plasma vitamin C and overall CCMR.

Although previous studies have examined the associations between quantity of F&V intake and CVD,^23^ few have done so in a population of people with diabetes with an extended duration of follow-up,^24, 25^ and none have examined the association using plasma vitamin C as a biomarker of F&V intake. Among 10 000 individuals with diabetes who were followed up for nine years in the European Prospective Investigation into Cancer and Nutrition (EPIC) study, each 80 g increase in self-reported F&V intake (including legumes) was associated with a hazard ratio (HR) of 0.88 (95% CI: 0.81, 0.95) for CVD mortality, whereas each 80 g increase in fruit was associated with an HR of 0.90 (0.81, 0.91).^24^ In contrast, among 1400 Japanese adults with diabetes who were followed up for 8 years, Tanaka *et al.*^25^ did not find an association between self-reported F&V intake and incident coronary heart disease, although a protective effect on incident stroke was reported. However, because F&V intake was assessed only at baseline in both studies, the potential benefits of increases in intake could not be examined.

To our knowledge, only two other studies have investigated associations between variety of F&V intake and CVD risk, and although they were both in non-diabetes-specific populations neither was able to find an association with incident coronary heart disease, despite sample sizes of 20 000 and 143 000 with follow-up durations of 10 and 20 years, respectively.^13, 12^

Consistent with our finding that increased fruit intake was associated with a smaller waist circumference over 1 year, Bertoia *et al.*^26^ show that each increase in daily serving of fruit and vegetable is associated with a 240 g and 110 g reduction in weight, respectively, over 4 years of follow-up. To put only this finding into clinical context, if everybody with newly developed diabetes were to increase their quantity of F&V intake by 250 g per day (1 s.d. in our study), they would experience an approximate reduction in waist circumference of 1 cm – the benefit of which would be a 2% reduction in the risk of cardiovascular event.^27^

Although the associations between plasma vitamin C levels and cardiovascular risk factor levels were not discrepant with those for quantity of F&V intake, there are several reasons that might explain why the associations were weaker for plasma vitamin C when the opposite might have been expected.^9^ First, because an increasing number of foods are enriched with vitamin C, F&V can no longer be assumed to be the main source of intake of this vitamin. Second, plasma levels of vitamin C plateau at the upper end of the normal range,^28^ meaning that any additional increase in intake will not be correctly reflected in plasma levels. Finally, a number of factors such as physical activity, BMI and the efficiency with which the body metabolises vitamin C, which is partially genetically determined, have all been associated with plasma vitamin C levels.^29^ Thus, to gain a better understanding of the association between F&V intake and cardiovascular risk factor levels, future studies should use a complementary approach in which several F&V biomarkers are used in combination, as has been done previously.^30^

Although the mechanisms by which F&V might improve cardiovascular risk factor levels are not yet fully understood, there are several plausible hypotheses. F&V provide an abundant source of vitamins, minerals and phytochemicals that could help reduce cardiovascular risk factor levels, by acting both alone and in synergy, by counteracting the potentially harmful effects of oxidative stress.^31^ A second explanation could be that because F&V generally have a low energy content any increase in intake could displace energy-dense foods from the diet,^32^ thereby aiding weight loss.

### Strengths and limitations

Our study has a number of important strengths, including the population-based study design, use of repeat measures of all exposures and outcomes over 5 years of follow-up and complementary analyses using plasma vitamin C as an objective biomarker of F&V intake. Although it is known that F&V intake reported using an FFQ generally leads to an overestimation of intake in comparison with a 7-day dietary recall questionnaire,^33^ an additional major strength of our study is that we used the FFQ across time points to estimate change in intake, for which it has been shown to be equally as valid as multiple 24-h recalls.^34^ However, as with any self-reported measure, FFQs may be vulnerable to recall and social desirability biases. We were also able to adjust for a wide range of potential confounders, reducing the likelihood that our findings are explained by confounding.

The limitations of our study also warrant discussion. Our analyses were limited to only 46% of the original ADDITION-Cambridge study population because of missing data at one or more of the follow-up clinic visits. Excluded participants reported having a lower intake of F&V, a larger waist circumference and higher HbA_1c_ levels at baseline. However, when we performed multiple imputation analyses the results were similar, suggesting that bias due to missing data is unlikely. In addition, because of the number of hypothesis tests conducted, statistically significant results may have occurred solely because of the play of chance. As the majority of our population is white and middle-aged, the generalisability of our findings to other ethnicities and age groups requires caution. Furthermore, because of the lack of heterogeneity in variety of F&V intake observed in our study cohort, we cannot rule out variety in intake as playing an important role in CVD – we therefore suggest that this be studied in future cohorts.

## Conclusions

Increased intake of F&V early in the course of diabetes is associated with improvements in a number of important cardiovascular risk factors. It will be beneficial to investigate why the early increases in F&V intake are not maintained in the longer term, and future research should focus on identifying strategies to help patients maintain improvements in diet.

## Figures and Tables

**Table 1:** Characteristics of the study population

	*Baseline*	*1 year*	*5 years*
*N*	401	–	–
Men (%)	227, 56.6%	–	–
Age (years)	61.4±6.6	62.5±6.6	66.6±6.7
Height (m)	168.0±9.4	167.7±9.4	167.1±9.5
Weight (kg)	93.1±17.4	90.0±17.4	89.7±18.1
Waist (cm)	110.4±13.2	107.6±12.9	107.0±13.6
SBP (mmHg)	141.5±20.1	135.2±19.1	134.6±16.5
HDL (mmol/l)	1.20±0.32	1.23±0.33	1.30±0.33
TG (mmol/l)	1.7 (1.2, 2.4)	1.6 (1.1, 2.3)	1.6 (1.2, 2.3)
HbA_1c_ (%)	6.7 (6.2, 7.3)	6.3 (5.9, 6.7)	6.8 (6.4, 7.4)
HbA_1c_ (mmol/mol)	49.7 (44.3, 56.3)	45.4 (41.0, 49.7)	50.8 (46.5, 57.4)
CCMR	−0.04±0.51	−0.3±0.5	−0.3±0.5
Glucose-lowering drugs (*n* %)	1, 0.2%	123, 30.7%	242, 60.3%
Blood-pressure-lowering drugs (*n* %)	288, 71.8%	324, 80.8%	329, 82.0%
Lipid-lowering drugs (*n* %)	117, 29.2%	298, 74.3%	339, 84.5%
			
*Smoking (*n *%)*			
Current	53, 13.2	50, 12.4	45, 11.2
Ex	198, 49.4	201, 50.1	200, 49.9
Never	150, 37.4	150, 37.4	156, 38.9
Alcohol (g/day)	4.68 (0.51, 11.33)	4.66 (0.00, 10.07)	1.55 (0.00, 9.10)
Energy intake (kcal/day)	1918.4±617.6	1681.6±528.7	1653.1±538.8
MVPA (mins/day)	72.1 (30.0, 146.3)	81.4 (31.6, 159.9)	74.4 (29.1, 148.7)
*Occupational social class*		–	–
Managerial and professional	144, 35.9%		
Intermediate	93, 23.2%		
Routine and manual	164, 40.9%		
Plasma vitamin C (μmol/l), *n*=277	55.0 (39.0, 68.7)	56.7 (40.6, 72.2)	58.0 (39.9, 70.9)
			
*Variety (number per week)*			
F&V	23.3±6.2	24.1±5.9	23.3±6.0
Fruit	6.7±2.6	7.0±2.4	6.6±2.5
Vegetable	16.6±4.5	17.1±4.2	16.7±4.3
*Quantity (g/day)*			
F&V	489.5 (336.2, 690.6)	555.1 (412.1, 746.7)	495.8 (365.7, 662.5)
Fruit	216.7 (110.9, 362.9)	259.0 (163.8, 399.7)	223.7 (112.1, 361.9)
Vegetable	262.5 (187.4, 340.2)	283.5 (201.9, 367.1)	264.0 (189.3, 350.8)

Abbreviations: CCMR, clustered cardiometabolic risk scores; F&V, fruits and vegetables; HbA_1c_, haemoglobin A1c; HDL, high-density lipoprotein; IQR, interquartile range; MVPA, moderate-to-vigorous physical activity; SBP, systolic blood pressure; TG, triglycerides. Values are presented as mean±s.d., *n*, % and median (IQR) – denotes that the values are the same as baseline.

**Table 2:** Associations between changes in quantity of F&V intake and changes in plasma vitamin C (per s.d.) with cardiometabolic risk factors at the relevant follow-up time point, presented as regression coefficients (and 95% confidence intervals) per 1 s.d. increase in the exposure, estimated from linear mixed models

	*Baseline to 1 year*		*1–5 years*	
	*Model 1*	*Model 2*	*Model 1*	*Model 2*
*Waist (cm)*				
Plasma vitamin C	−0.66 (−1.51, 0.20)	−0.62 (−1.31, 0.08)	−0.31 (−1.13, 0.52)	−0.50 (−1.25, 0.25)
Quantity of F&V	−0.59 (−1.32, 0.13)	−0.92 (−1.57, −0.27)	0.12 (−0.60, 0.85)	−0.09 (−0.78, 0.60)
Quantity of fruit	−0.69 (−1.45, 0.07)	−0.89 (−1.56, −0.23)	0.17 (−0.59, 0.93)	−0.28 (−0.96, 0.40)
Quantity of vegetable	−0.18 (−0.88, 0.53)	−0.48 (−1.13, 0.16)	0.06 (−0.64, 0.77)	0.23 (−0.47, 0.93)
				
*SBP (mm Hg)*				
Plasma vitamin C	−1.27 (−2.98, 0.44)	−1.36 (−3.09, 0.37)	−0.37 (−2.21, 1.47)	−0.99 (−2.86, 0.88)
Quantity of F&V	−0.64 (−2.24, 0.95)	−0.73 (−2.36, 0.90)	0.38 (−1.21, 1.97)	0.31 (−1.43, 2.05)
Quantity of fruit	−0.26 (−1.87, 1.34)	0.44 (−1.23, 2.10)	0.70 (−0.90, 2.30)	0.89 (−0.81, 2.59)
Quantity of vegetable	−0.90 (−2.49, 0.68)	−1.89 (−3.48, −0.29)	−0.23 (−1.81, 1.35)	−0.62 (−2.36, 1.11)
				
*HbA*_*1c*_*(%)*				
Plasma vitamin C	−0.00 (−0.09, 0.08)	0.01 (−0.08, 0.10)	−0.04 (−0.14, 0.05)	−0.08 (−0.18, 0.01)
Quantity of F&V	−0.11 (−0.19, −0.03)	−0.11 (−0.20, −0.03)	0.01 (−0.07, 0.09)	−0.01 (−0.08, 0.10)
Quantity of fruit	−0.11 (−0.19, −0.03)	−0.12 (−0.20, −0.03)	0.01 (−0.07, 0.09)	0.01 (−0.10, 0.07)
Quantity of vegetable	−0.05 (−0.13, 0.03)	−0.05 (−0.13, 0.03)	0.01 (−0.07, 0.09)	0.04 (−0.05, 0.12)
				
*TG (mmol/l)*				
Plasma vitamin C	0.01 (−0.08, 0.10)	−0.01 (−0.10, 0.08)	−0.08 (−0.18, 0.01)	−0.11 (−0.21, −0.01)
Quantity of F&V	0.01 (−0.09, 0.08)	−0.05 (−0.14, 0.03)	−0.07 (−0.15, 0.02)	−0.09 (−0.18, 0.00)
Quantity of fruit	−0.05 (−0.14, 0.03)	−0.08 (−0.17, 0.00)	−0.06 (−0.15, 0.02)	−0.10 (−0.19, −0.01)
Quantity of vegetable	0.07 (−0.01, 0.15)	0.03 (−0.06, 0.11)	−0.03 (−0.12, 0.05)	−0.03 (−0.12, 0.06)
				
*HDL-c (mmol/l)*				
Plasma vitamin C	0.00 (−0.02, 0.03)	0.02 (−0.01, 0.04)	0.03 (0.00, 0.05)	0.04 (0.01, 0.06)
Quantity of F&V	−0.01 (−0.03, 0.01)	−0.01 (−0.03, 0.01)	0.03 (0.01, 0.06)	0.04 (0.01, 0.06)
Quantity of fruits	0.00 (−0.03, 0.02)	−0.00 (−0.02, 0.02)	0.03 (0.00, 0.05)	0.03 (0.01, 0.05)
Quantity of vegetables	−0.02 (−0.04, 0.00)	−0.01 (−0.03, 0.01)	0.02 (0.00, 0.05)	0.02 (0.00, 0.05)
				
*CCMR*				
Plasma vitamin C	−0.02 (−0.07, 0.02)	−0.04 (−0.08, 0.00)	−0.05 (−0.09, 0.00)	−0.07 (−0.12, −0.03)
Quantity of F&V	−0.03 (−0.07, 0.01)	−0.04 (−0.08, −0.01)	−0.02 (−0.06, 0.01)	−0.03 (−0.07, 0.01)
Quantity of fruit	−0.04 (−0.08, 0.00)	−0.04 (−0.08,−0.01)	−0.01 (−0.05, 0.03)	−0.04 (−0.08, 0.00)
Quantity of vegetable	0.01 (−0.03, 0.05)	−0.02 (−0.06, 0.02)	−0.02 (−0.06, 0.02)	−0.01 (−0.05, 0.03)

Abbreviations: CCMR, clustered cardiometabolic risk score; F&V, fruits and vegetables; HbA_1c_, haemoglobin A1c; HDL, high-density lipoprotein; SBP, systolic blood pressure; TG, triglycerides. Model 1: adjusted for age and sex.

Model 2: model 1+intervention group, occupational socio-economic status, baseline and follow-up smoking status, physical activity, alcohol intake, total energy intake (except plasma vitamin C), blood pressure-lowering (for SBP and CCMR), glucose-lowering medication (for HbA_1c_ and CCMR) and lipid-lowering medication (for TG, HDL-c and CCMR) and change in variety of intake (except plasma vitamin C). Between baseline and 1 year, one s.d. plasma vitamin *C*=23.40 μmol/l; quantity F&V=249.91 g; quantity of fruit=191.61 g; quantity of vegetable=151.28 g. Between 1 and 5 years, one s.d. plasma vitamin *C*=22.54 μmol/l; quantity of F&V=270.05 g; quantity of fruit=196.66 g; quantity of vegetables=131.54 g.

**Table 3:** Associations between changes in variety of F&V intake (per s.d.) and cardiometabolic risk factors at the relevant follow-up time point, presented as regression coefficients (and 95% confidence intervals) per 1 s.d. increase in the exposure, estimated from linear mixed models

	*Baseline to 1 year*		*1–5 years*	
	*Model 1*	*Model 2*	*Model 1*	*Model 2*
*Waist (cm)*				
Variety of F&V	0.18 (−0.51, 0.88)	0.18 (−0.46, 0.83)	0.20 (−0.50, 0.89)	0.53 (−0.12, 1.18)
Variety of fruit	−0.46 (−1.16, 0.25)	0.09 (−0.59, 0.76)	0.45 (−0.26, 1.15)	0.42 (−0.23, 1.07)
Variety of vegetable	0.56 (−0.13, 1.25)	0.23 (−0.41, 0.88)	−0.03 (−0.68, 0.69)	0.42 (−0.24, 1.07)
				
*SBP (mm Hg)*				
Variety of F&V	−0.59 (−2.17, 1.00)	−0.60 (−0.21, 1.01)	−0.00 (−1.58, 1.58)	−0.17 (−1.79, 1.45)
Variety of fruit	−0.79 (−2.38, 0.79)	−0.52 (−2.20, 1.15)	−0.16 (−1.43, 1.74)	−0.19 (−1.81, 1.43)
Variety of vegetable	−0.24 (−1.81, 1.34)	−0.41 (−2.02, 1.19)	−0.11 (−1.69, 1.46)	0.13 (−1.50, 1.75)
				
*HbA*_*1c*_*(%)*				
Variety of F&V	−0.05 (−0.13, 0.03)	−0.05 (−0.13, 0.04)	0.07 (−0.01, 0.15)	0.07 (−0.01, 0.16)
Variety of fruit	−0.07 (−0.15, 0.01)	−0.03 (−0.12, 0.06)	0.07 (−0.01, 0.15)	0.06 (−0.02, 0.14)
Variety of vegetable	−0.01 (−0.09, 0.07)	−0.05 (−0.13, 0.04)	0.05 (−0.03, 0.13)	0.05 (−0.03, 0.14)
				
*TG (mmol/l)*				
Variety of F&V	−0.01 (−0.09, 0.07)	0.00 (−0.08, 0.08)	0.03 (−0.06, 0.11)	0.06 (−0.02, 0.15)
Variety of fruit	−0.04 (−0.13, 0.04)	−0.02 (−0.11, 0.06)	0.01 (−0.07, 0.09)	0.04 (−0.05 0.12)
Variety of vegetable	0.02 (−0.07, 0.10)	0.02 (−0.07, 0.10)	0.03 (−0.05, 0.11)	0.05 (−0.03, 0.13)
				
*HDL-c (mmol/l)*				
Variety of F&V	0.01 (−0.03, 0.02)	−0.01 (−0.03, 0.01)	0.00 (−0.03, 0.02)	−0.01 (−0.03, 0.01)
Variety of fruit	0.01 (−0.01, 0.03)	−0.01 (−0.04, 0.01)	−0.00 (−0.03, 0.02)	−0.01 (−0.03, 0.01)
Variety of vegetable	−0.02 (−0.04, 0.01)	−0.00 (−0.03, 0.02)	0.00 (−0.02, 0.02)	−0.01 (−0.03, 0.01)
				
*CCMR*				
Variety of F&V	−0.01 (−0.05, 0.03)	0.00 (−0.03, 0.04)	0.01 (−0.03, 0.05)	0.02 (−0.01, 0.06)
Variety of fruit	−0.05 (−0.09, −0.01)	−0.00 (−0.04, 0.04)	0.02 (−0.01, 0.06)	0.02 (−0.02, 0.06)
Variety of vegetable	0.03 (−0.01, 0.07)	0.01 (−0.03, 0.05)	0.00 (−0.04, 0.04)	0.02 (−0.02, 0.06)

Abbreviations: CCMR, clustered cardiometabolic risk scores; F&V, fruits and vegetables; HbA_1c_, haemoglobin A1c; HDL, high-density lipoprotein; SBP, systolic blood pressure; TG, triglycerides. Model 1: adjusted for age and sex.

Model 2: model 1+intervention group, occupational socio-economic status, baseline and follow-up smoking status, physical activity, alcohol intake, blood pressure-lowering (for SBP and CCMR), glucose-lowering medication (for HbA_1c_ and CCMR) and lipid-lowering medication (for TG, HDL-c and CCMR) and change in quantity of intake.

Between baseline and 1 year, one s.d. variety of fruit and vegetables=4.3 items; variety of fruit=2.3 items; variety of vegetables=3.1 items. Between 1 and 5 years, one s.d. variety fruit and vegetables=4.6 items; variety of fruit=2.2 items; variety of vegetables=3.3 items.
