# Supplementary material for: Fruit and vegetable intake and cardiovascular risk factors in people with newly diagnosed type 2 diabetes
Source: Eur J Clin Nutr. 2016 Oct 19;71(1):115–21. doi: 10.1038/ejcn.2016.180 (PMC5218580; doi:10.1038/ejcn.2016.180)
Supplement: Supplementary Figure [file ejcn2016180x1.docx]

**Supplementary figure 1: ADDITION-Cambridge analysis population flowchart**
